# Multicomponent reactions III

**DOI:** 10.3762/bjoc.15.192

**Published:** 2019-08-20

**Authors:** Thomas J J Müller

**Affiliations:** 1 Heinrich-Heine Universität Düsseldorf, Institut für Organische Chemie und Makromolekulare Chemie, Universitätsstraße 1, D-40225 Düsseldorf, Germany

**Keywords:** MCR, multicomponent reactions

In times of steadily increasing relevance of sustainability and environmental concern, the concept of multicomponent reactions (MCRs) [1] has become a particularly powerful principle of synthetic design, combining synthetic efficiency with conceptual efficacy. The importance of rapid lead finding and identification has demanded novel ways of synthetic approaches, ultimately approaching the ideal synthesis [2–3]. As an evergreen in organic chemistry MCRs never became old-fashioned or tedious, because they always inspire creative spirits by following the fundamental quest: more than two compounds are reacted in a one-pot fashion to form two or more bonds. This fundamental principle, recognized by Ugi's groundbreaking developments in isonitrile-based chemistry directly leads to one-pot methodologies as a reactivity-based concept [4]. Reactive functionalities that are repetitively being generated and transformed represent the basis of the underlying general principle. Therefore, MCRs are equally intriguing for industrial applications as exciting and stimulating for academia, especially, for approaching new shores of interdisciplinarity by concatenating elementary steps to new sequences and ultimately to complex functional molecules in a one-pot fashion.

This thematic issue on multicomponent reactions proceeds the previously released issues from 2011 and 2014 [5–6]. Moreover, by the majority of the contributing authors it also becomes a vivid testimony of last year's 7th International Conference on Multicomponent Chemistry and Related Reactions that was held in Düsseldorf, Germany [7]. All contributions in this issue report or summarize recent developments of this highly dynamic field in a snap shot fashion. The agenda broadly spans over modern synthetic chemistry, from isonitrile and condensation-based MCRs over metal-catalyzed and mediated sequences to algorithms of synthetic efficiency. Biologically and pharmaceutically relevant scaffolds are likewise tackled as chromophores, methodology development and conceptual design of macro(hetero)cycles go hand in hand with MCR-based heterocyclic chemistry. As in the previous thematic issues also this issue opens the actual field of MCR chemistry to a broader interested community by five reviews on MCR based concepts.

As the guest editor of this thematic issue in the *Beilstein Journal of Organic Chemistry* I cordially thank all authors, dedicated and excellent scientists, for sharing their exciting findings and, in particular, I am grateful to the staff of the Beilstein-Institut for their excellent support and professional realization.

Thomas J. J. Müller

Düsseldorf, July 2019

## References

[1] Zhu J, Wang Q, Wang M (2014). Multicomponent Reactions in Organic Synthesis.

[2] Wender P A, Handy S T, Wright D L (1997). Chem Ind.

[3] Gaich T, Baran P S (2010). J Org Chem.

[4] Müller T J J, Müller T J J (2014). 1. General Discussion and Reactions Involving a Carbonyl Compound as Electrophilic Component. Multicomponent Reactions.

[5] (2019). https://www.beilstein-journals.org/bjoc/series/16.

[6] (2019). https://www.beilstein-journals.org/bjoc/series/39.

[7] Müller T J J, Schaper K (2019). Nachr Chem.

